# Fibrin clot strength is associated with increased risk of major adverse cardiac events after TAVR

**DOI:** 10.1007/s00392-025-02749-7

**Published:** 2025-10-06

**Authors:** David Hesselbarth, Michelle D’Orazio, Giovanni Ciccarone, Diona Gjermeni, Carina Jülch, Marius Wessinger, Mariya Maslarska, Jonathan Rilinger, Ingo Hilgendorf, Dennis Wolf, Klaus Kaier, Daniel Duerschmied, Torben Pottgiesser, Constantin von zur Mühlen, Dirk Westermann, Christoph B. Olivier

**Affiliations:** 1 Department of Cardiology and Angiology, University Heart Center Freiburg-Bad Krozingen, Faculty of Medicine, University of Freiburg, Hugstetter Str. 55, 79106 Freiburg, Germany; 2 Institute of Medical Biometry and Statistics, Faculty of Medicine and Medical Center, University of Freiburg, Freiburg, Germany; 3 Department of Cardiology, Angiology, Haemostaseology and Medical Intensive Care, Medical Faculty Mannheim, University Medical Centre Mannheim, Heidelberg University, Heidelberg, Germany; 4 European Center for AngioScience (ECAS) and German Center for Cardiovascular Research (DZHK) Partner Site Heidelberg/Mannheim, Mannheim, Germany

**Keywords:** Transcatheter aortic valve replacement, TAVR, Hemostatic markers, Thrombelastography, TEG

## Abstract

**Background:**

Patients after transcatheter aortic valve replacement (TAVR) are at increased risk of both major adverse cardiac events (MACE), including ischemic and thrombotic complications, as well as significant bleeding. Given this delicate balance between prothrombotic and hemorrhagic risk, the assessment of hemostatic markers might help identify patients at increased risk.

**Aim:**

To identify hemostatic markers associated with MACE and bleeding following TAVR.

**Methods:**

In this prospective single-center cohort study, of patients undergoing TAVR from November 2020 to June 2022, the association of hemostatic profiles and clinical outcomes was assessed. The profiling included thromboelastography (TEG), light transmission aggregometry (LTA), and conventional laboratory markers to assess thrombogenicity. The outcome was MACE (death, myocardial infarction, or stroke) and major/non-major clinically relevant bleeding at 6 months.

**Results:**

Of the 107 patients included, 104 completed follow-up. At 6 months, 9% experienced MACE, and 10% had clinically relevant bleeding. Platelet–fibrin clot strength, reflected by the maximum amplitude (MA-citrated kaolin) in thrombelastography, was elevated in patients with MACE (per 1 mm increase: HR 1.26 [1.02;1.56], *p* = 0.03). Fibrin’s role in the maximum clot strength was crucial. Elevated fibrinogen levels, increased citrated functional fibrinogen (MA-CFF), and faster fibrin formation (alpha-angle[α]) associated with a higher risk of MACE (per 20 mg/dL fibrinogen increase: HR 1.15 [1.02–1.28], *p* = 0.02; per 5 mm MA-CFF increase: HR 1.59 [1.12–2.26], *p* < 0.01; per degree α-citrated rapid TEG increase: HR 1.55 [1.10–2.19], *p* = 0.01), respectively. High on-treatment ADP-induced platelet reactivity assessed by LTA was associated with a lower risk of major and non-major clinically relevant bleeding at 6 months (per 10% MA-ADP increase: HR 0.66 [0.47;0.93]﻿ *p=0.02* ﻿﻿).

**Conclusion:**

Selected hemostatic markers associated with the risk of MACE and bleeding within 6 months in patients post-TAVR, with fibrin clot strength identified as a principal marker.

**Graphical Abstract:**

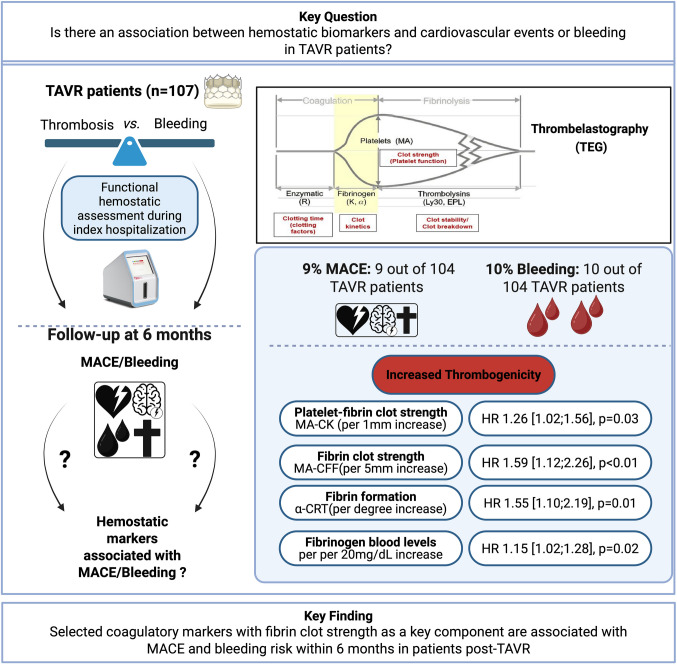

**Supplementary Information:**

The online version contains supplementary material available at 10.1007/s00392-025-02749-7.

## Introduction

Patients undergoing transcatheter aortic valve replacement (TAVR) are typically elderly and often present with significant comorbidities, including coronary artery disease, peripheral artery disease, or atrial fibrillation. These conditions increase their risk for both ischemic and bleeding events, requiring careful consideration of antithrombotic therapy. Reported incidences of adverse events post-TAVR are approximately 7% for ischemic stroke, 10% for all-cause mortality, and 15% for major bleeding within 1 year [1].

Given the high rates of ischemic, and bleeding events in patients after TAVR, identifying biomarkers for risk stratification and prediction is crucial. While biomarkers of myocardial damage, heart failure, and inflammation are associated with cardiovascular events and poorer outcomes post-TAVR, data on hemostatic markers for predicting these events are limited [2]. Antithrombotic management in TAVR patients is challenging, and routine anticoagulation for all patients has not proven clinical benefit [3, 4]. Identifying hemostatic markers may help optimize and individualize antithrombotic therapy.

Conventional coagulation tests are limited by their in vitro nature and do not fully capture the in vivo coagulation system. Functional coagulation tests such as light transmission aggregometry (LTA) and thrombelastography (TEG) have shown promising results in predicting cardiovascular events in cardiac patients [5, 6].

This analysis aimed to identify hemostatic markers associated with 6-month post-TAVR clinical outcomes and to enhance risk stratification and the prediction of cardiovascular events and bleeding in TAVR patients.

## Methods

### Study design and cohort

RISTRATAVI was an observational, prospective single-center cohort study, that assessed the hemostatic profiles of patients undergoing TAVR during their index hospitalization (days 1–17 post-TAVR, median day 4 (IQR 3–6)). Blood samples were obtained as close to hospital discharge as possible. The study design has been previously described (NCT03649594). The clinical outcome was a composite of death, myocardial infarction, stroke/TIA (major adverse cardiovascular events, MACE), and major or clinically relevant non-major bleeding at 6 months. Patients undergoing TAVR were enrolled during hospitalization, excluding those with severe renal insufficiency (GFR < 30 ml/min/1.73 m^2^), severe thrombocytopenia (< 50 × 10^3^/µl), or undergoing valve-in-valve TAVR (Table S1). These exclusion criteria were used to minimize potential bias in coagulation and bleeding risk assessments. Patients were treated with single antiplatelet therapy, dual antiplatelet therapy, or anticoagulation as per standard of care. Study procedures adhered to the principles of the Helsinki Declaration and were approved by the ethics committee of the Albert-Ludwigs-University Freiburg, Germany.

### Functional hemostatic assessment and blood samples

Venous blood was collected using a 21-G butterfly needle during index hospitalization and was used for standard laboratory tests and functional hemostatic assessment. A comprehensive list of the assessed hemostatic and inflammatory parameters is provided in Table S4. Functional hemostatic assessment was performed with thrombelastography TEG® 6 s Hemostasis Analyzer (Haemonetics® Corp., Boston, MA, USA) and light transmission aggregometry using the Platelet Aggregation Profiler PAP-8E (Bio/Data Corp., Horsham, PA, USA) following standard manufacturer protocols as previously described [7]. Key TEG parameters included the maximum amplitude (MA), which evaluates the overall platelet–fibrin clot strength when activated by citrated kaolin (CK), CK with heparinase (CKH), or citrated rapidTEG (CRT). When activated by citrated functional fibrinogen (CFF), MA-CFF specifically measures the isolated fibrin contribution to clot strength. Activation of the thromboxane pathway using arachidonic acid (AA) reflected the platelet contribution to clot strength (AA-MA). R-Time measures the time to initial clot formation comparable to INR and aPTT evaluated by standard laboratory tests. Key LTA parameters included primary and maximum aggregation (PA, MA) as well as disaggregation (DA) induced by arachidonic acid (AA) or adenosine diphosphate (ADP).

### Outcomes and follow-up

The outcomes were a composite of death, myocardial infarction, or stroke/TIA (MACE) and non-procedure related major (VARC types 2–4) or clinically relevant non-major bleeding (VARC types 1–2). Follow-up was performed at 6 months ± 12 weeks. All suspected events were evaluated by three independent board-certified specialists in internal medicine, based on the updated valve academic research consortium 3 (VARC-3) definitions [8]. Discrepancies were resolved by a board-certified cardiologist.

### Statistical considerations

Categorical variables were presented as numbers and frequencies and continuous variables as medians with interquartile range (IQR). Cox regression was used for the time-to-event analysis for the composite outcome of MACE and bleeding events. ROC analysis was used to assess the discriminative capacity of different hemostatic markers, and the optimal cutoff was determined. Patients were then stratified based on this cutoff, and survival analysis was conducted using the Kaplan–Meier method. Two-tailed tests with *p* values ≤ 0.05 were considered significant. Data were analyzed using Prism 10.2.0 (GraphPad) and SPSS 29.0.0.1.

## Results

### Patient population

**Fig. 1:**
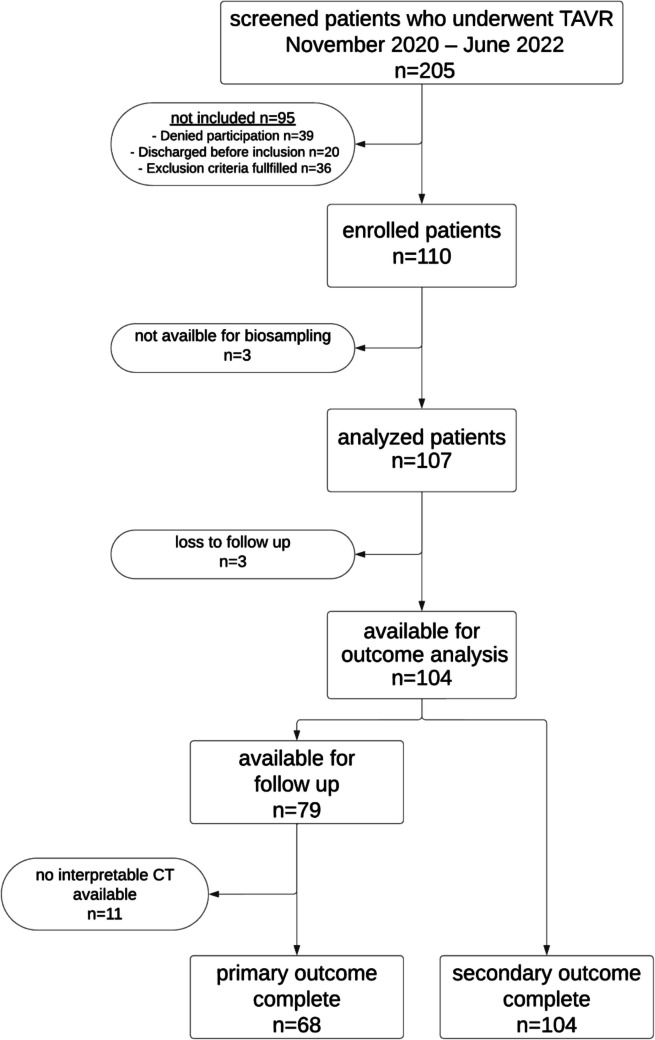
Flowchart of the patient selection

Between November 2020 and June 2022, 205 patients were screened and 107 were enrolled in this single-center observational prospective cohort study within 17 days post-TAVR (Fig. 1). Clinical follow-up for the secondary outcome was completed in 104 patients. Baseline characteristics are provided in Table 1. The median age was 82 (interquartile range, IQR 79–85) years, with 55 (51%) males. The median time to functional hemostatic measurement after blood draw was 33 [30;40] min for TEG and 75 [40;100] min for LTA. Details about the parameters measured are provided in Table S4.

**Table 1:** Clinical baseline characteristics

*Baseline characteristics*	Total *n* = 107
Demography	
Age [years]	82 (79–85)
Male	55 (51%)
BMI [kg/m^2^]	25.1 (23.3–27.3)
Patient medical history	
CHA_2_DS_2_-VASc score	5 (4–6)
HAS-BLED	3 (2–4)
Atrial fibrillation	56 (52%)
TIA/stroke	21 (20%)
Myocardial infarction	12 (11%)
LVEF [%]	55 (50–60)
History of bleeding	13 (12%)
History of VTE	10 (9%)
Coronary artery disease	76 (71%)
PAD	9 (8%)
Chronic kidney failure	32 (30%)
History of cancer	22 (21%)
Arterial hypertension	103 (96%)
Diabetes mellitus	25 (23%)
Smoking	16 (15%)
Positive family history	11 (10%)
Procedural characteristics	
Balloon expandable valve	93 (87%)
Self-expanding valve	14 (13%)
Antithrombotic therapy at discharge	
SAPT	27 (25%)
DAPT	33 (31%)
OAC ± APT	47 (44%)

Values expressed in median (Interquartile range [IQR]) or *n* (%)

*BMI*, body mass index; *TIA*, transient ischemic attack; *LVEF*, left ventricular ejection fraction; *VTE*, venous thromboembolism; *PAD*, peripheral artery disease; *SAPT*, single antiplatelet therapy; *DAPT*, dual antiplatelet therapy; *OAC*, oral anticoagulation; *APT*, Antiplatelet therapy

### Hemostatic markers as predictors for clinical outcomes

**Fig. 2:**
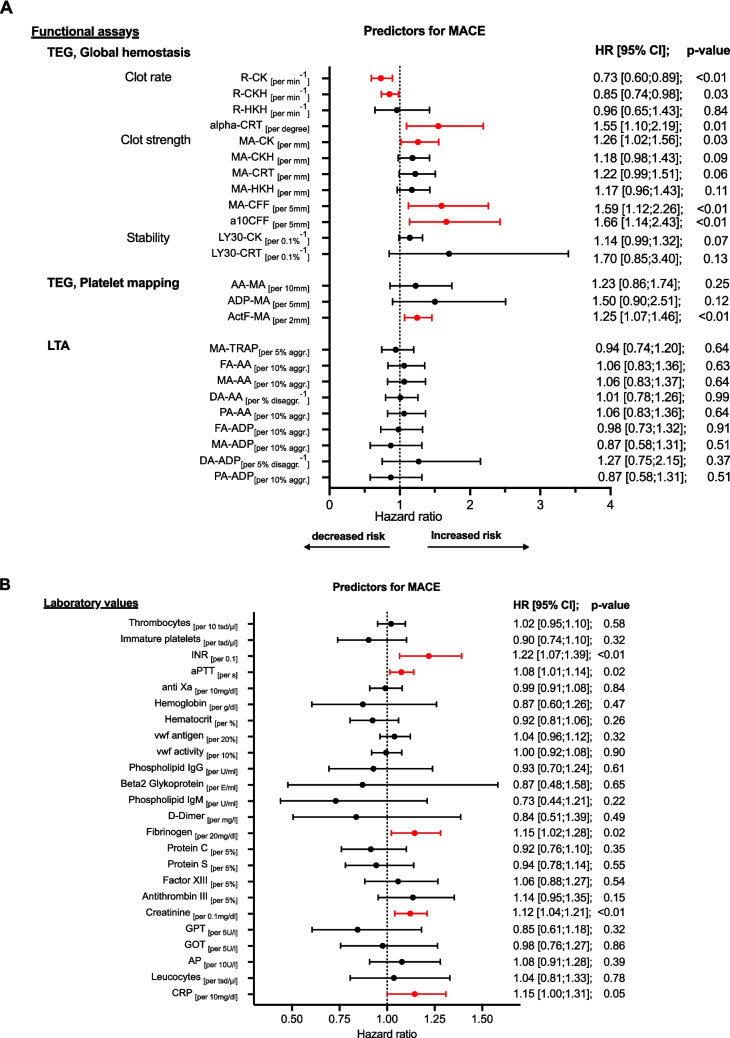
**A**, **B** Hemostatic markers associated with the occurrence of MACE at 6 months. COX regression with functional assays (**A**) and laboratory values (**B**) measured after TAVR during index hospitalization. HALT was diagnosed with CT scan 6 months post-TAVR. Increased risk indicates hypercoagulability. Decreased risk indicates hypocagulabilty. MACE, major adverse cardiac event; HR, hazard ratio; TEG, thrombelastography; GH, global hemostasis; PM, platelet mapping; LTA, light transmission aggregometry; OR, odds ratio R: time to initial clot formation, alpha: kinetics of clot formation; MA, maximum aggregation; a10CFF, firmness of fibrin 10 min after start of test; Ly30, thrombolysis 30 min after reaching MA; CK, citrated kaolin; CKH, kaolin + heparinase; CRT, kaolin + tissue factor; HKH, heparinized kaolin with heparinase; CFF, citrated functional fibrinogen; ActF, reptilase + factor XIII.; AA, arachidonic acid; ADP, adenosine diphosphate; TRAP, thrombin receptor activating peptide; INR, international normalized ratio; vwf, von Willebrand Faktor

MACE occurred in 9 out of 104 patients (9%) (Table 2). TEG-measured citrated kaolin-induced maximum amplitude (MA-CK) was elevated in patients with MACE, indicating increased hypercoagulability (per 1 mm increase MA-CK: HR 1.26 [1.02;1.56], *p* = 0.03). Fibrin’s role in the maximum clot stability was crucial, with elevated fibrinogen levels, increased fibrin clot strength (MA-CFF), and faster fibrin formation (alpha-CRT) all linked to a higher risk of MACE (per 20 mg/dL fibrinogen increase: HR 1.15 [1.02–1.28], *p* = 0.02; per 5 mm MA-CFF increase: HR 1.59 [1.12–2.26], *p* < 0.01; per degree alpha-CRT increase: HR 1.55 [1.10–2.19], *p* = 0.01). Platelet aggregation activated via thromboxane A2 showed no significant association with MACE (per 10 mm increase AA-MA: HR 1.23 [0.86;1.74], *p* = 0.25, Fig. 2A).

**Table 2:** Secondary outcomes

*Secondary outcome*	Total *n* = 104
MACE Death Myocardial infarction TIA/stroke	9 (9%) 4 (4%) 1 (1%) 5 (5%)
Bleeding events VARC type 1 VARC type 2 VARC type 3 Not classified in VARC	25 (24%) 4 (4%) 2 (2%) 4 (4%) 15 (14%)

Values expressed in *n* (%)

*HALT*, hypoattenuated leaflet thickening; *MACE*, major adverse cardiovascular events; *TIA*, transient ischemic attack; *VARC*, Valve Academic Research Consortium

**Fig. 3:**
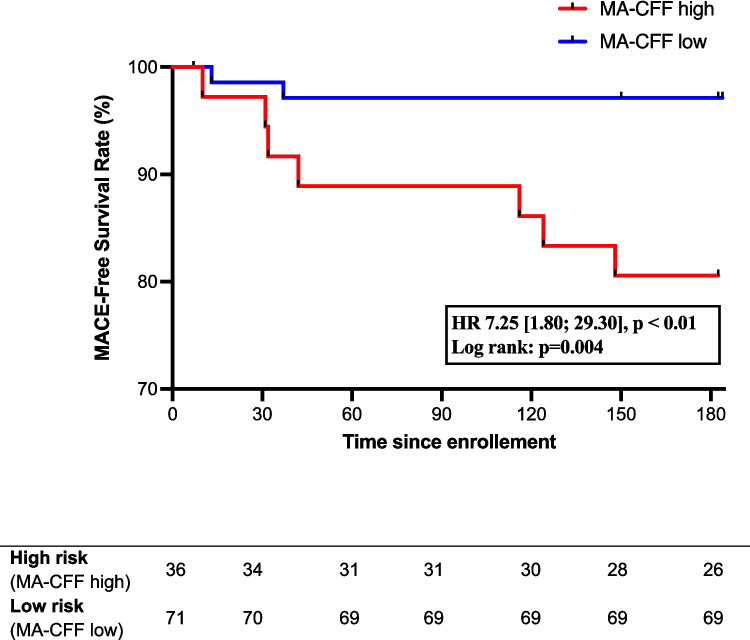
Kaplan–Meier for MACE (abbreviations see Fig. 2)

The optimal cutoff for MA-CFF in predicting MACE was determined to be 34.85 mm by ROC analysis, demonstrating good discriminatory ability (sensitivity 78%, specificity 70%, AUC 0.74 [0.55; 0.93], *p* = 0.02). Patients were grouped into MA-CFF high (> 34.85 mm) and MA-CFF low (< 34.85 mm). In the high MA-CFF group, 7 out of 36 patients (19.4%) experienced MACE, compared to 2 out of 71 (2.9%) in the low MA-CFF group. In a Kaplan–Meier analysis, high MA-CFF was significantly associated with an increased risk for MACE (HR 7.25 [1.80; 29.30], *p* < 0.01; Fig. 3). Fibrinogen and MA-CFF correlated significantly (*r* = 0.68, [0.57;0.77], *p* < 0.01, *n* = 106).

Hypocoagulability marked by prolonged r-time in TEG along with increased INR and aPTT—indicating delayed thrombin generation in both intrinsic and extrinsic pathways—were significantly associated with MACE (per 1 min increase R-CKH: HR: 1.17 [1.02;1.35], *p* = 0.03; per 0.1 increase INR: HR 1.22 [1.07;1.39], *p* < 0.01 and per 1-s increase aPTT HR: 1.08 [1.01;1.14]; *p* = 0.02, Fig. 2).

Impaired renal function and increased inflammation were also linked to MACE (per 0.1 mg/dl increase creatinine: HR 1.12 [1.04;1.21], *p* < 0.01; per 10 mg/dl increase CRP: HR 1.15 [1.00;1.31], *p* = 0.05; Fig. 2B).

**Fig. 4:**
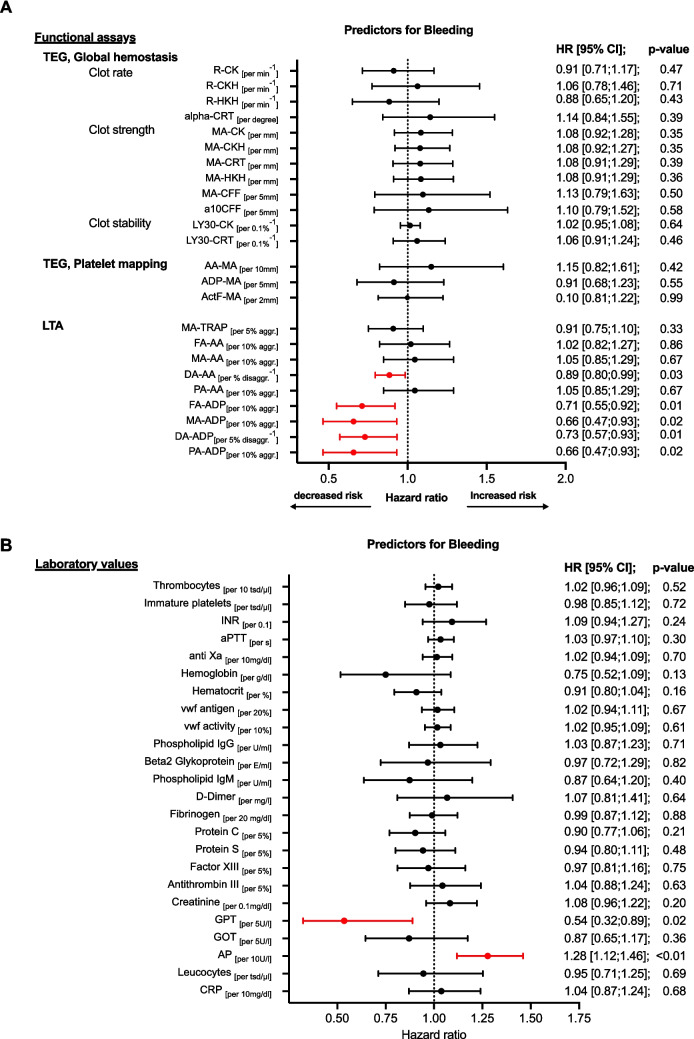
**A**, **B** Hemostatic markers associated with the occurrence of bleeding within 6 months. COX regression with functional assays (**A**) and laboratory values (**B**) measured after TAVR during index hospitalization. Increased risk indicates hypercoagulability. Decreased risk indicates hypocagulabilty bleeding: major and non-major clinically relevant bleeding, FA, final aggregation; DA, disaggregation; PA, primary aggregation. Other abbreviations see Figs. 2 and 3

Bleeding events occurred in 25 (24%) patients, with clinically relevant bleeding (VARC types 1–3) in 10 patients (10%) (Table 2, Figure S3). Those with higher maximum and primary ADP-induced aggregation in LTA had a significant lower risk of life-threatening or clinically relevant bleeding (per 10% increase MA-ADP: HR 0.66 [0.47;0.93], *p* = 0.02; per 10% increase PA-ADP: HR 0.66 [0.47;0.93], *p* = 0.02). A less rapid disaggregation response to both arachidonic acid (AA) and ADP was associated with a lower bleeding risk. (per 1% decrease DA-AA: HR 0.89 [0.80; 0.99], *p* = 0.03; per 5% decrease DA-ADP: HR: 0.73 [0.57;0.93], *p* = 0.01; Fig. 4A, B).

Subgroup analysis excluding patients not treated with an ADP-receptor antagonists such as Clopdiogrel or Ticagrelor showed consistent results, indicating that high on-treatment reactivity by ADP decreases the risk of clinically relevant bleeding per 10% increase MA-ADP: HR 0.38 [0.19; 0.75], *p* < 0.01 (Figure S2).

## Discussion

In this study, the prognostic value of a comprehensive panel of blood markers and functional hemostatic parameters in predicting the occurrence of HALT, cardiovascular events, and bleeding in patients after TAVR was assessed. The main findings were: (1) Overall platelet–fibrin clot stability assessed by TEG was associated with MACE. (2) Fibrin was a key contributor to overall clot stability. (3) High on-treatment platelet reactivity to ADP was associated with a lower incidence of clinically relevant bleeding.

Current risk scores for patients after TAVR primarily focus on clinical data [9], but biomarkers indicating myocardial injury and increased inflammation have also been linked to poor post-TAVR clinical outcomes [2]. To improve current prediction models, it has been proposed to incorporate new biomarkers into the existing risk scores [10]. TEG revealed increased clot strength in post-TAVR patients [11], but to date, no studies have explored the association of TEG biomarkers with clinical outcomes after TAVR. In this cohort, we identified that elevated MA-CK was associated with MACE (Fig. 3A), a finding consistent with previous studies in patients undergoing percutaneous coronary intervention (PCI) [12].

Fibrin-based clot formation (MA-CFF) emerged as a crucial determinant for overall clot stability. Patients with high MA-CFF (> 34.85 mm) had a 19.4% risk of MACE at 6 months (Fig. 4). In contrast, platelet aggregation activated via thromboxane A2 was not significantly associated with MACE. Elevated fibrinogen levels have been linked to cardiovascular disease in previous studies [13], and our group recently demonstrated their association with ischemic events in PCI patients with atrial fibrillation [14]. Additionally, a recent study found a strong correlation between fibrinogen and MA in both cardiovascular and COVID patients, suggesting its role as a key driver of arterial thrombosis [15]. Similarly, we observed a strong correlation between fibrinogen and MA-CFF, highlighting its role as a key factor for ischemic events. Elevated CRP and creatinine levels were also associated with MACE, consistent with previous findings [2]. Studies have shown that hypercoagulability increases in the early days following TAVR [11, 16]; however, a concomitant decline in platelet count is also frequently observed [17]. The precise dynamics and duration of these hemostatic changes remain poorly defined but are crucial for guiding individualized antithrombotic therapy. Routine anticoagulation in unselected TAVR patients has not demonstrated clinical benefit [3, 4]. However, selected patients with a procoagulant profile may benefit from targeted therapy during the period of post-TAVR hypercoagulability and elevated thrombotic risk. A more granular characterization of temporal dynamics of post-TAVR hypercoagulability may help to identify the period of highest thrombotic risk and thereby inform the design of a randomized controlled trial to evaluate treatment modification..

Prolonged r-time assessed by TEG, along with increased INR and aPTT, was associated with MACE, indicative of severely sick patients with deranged coagulation. In such cases, intensified antithrombotic treatment may be of questionable benefit and warrants cautious consideration. Furthermore, differentiating between platelet driven aggregation and fibrin-mediated coagulation could be a valuable focus for future trials, potentially enabling a more personalized approach to antithrombotic therapy post-TAVR.

The routine use of dual antiplatelet therapy in post-TAVR patients is no longer recommended, as trials have shown an increased risk of bleeding without reducing ischemic events compared to single antiplatelet therapy [19]. This study further supports this recommendation, demonstrating that high on-treatment platelet reactivity to ADP, measured by LTA, was associated with a lower risk of major or non-major clinically relevant bleeding in TAVR patients. This finding was not confirmed by TEG measurement, consistent with previous reports indicating reduced sensitivity of TEG to ADP-mediated platelet activation [20]. The association between high on-treatment platelet reactivity and decreased bleeding risk has been previously demonstrated in other patient cohorts using various platelet mapping assays, supporting the development of a therapeutic window concept for personalized antithrombotic therapy [14, 21–23]. Data on optimal cutoff values for platelet function tests in the context of combined antiplatelet therapy and oral anticoagulation are limited [22].

### Strengths and limitations of the study

This is the first study to evaluate a comprehensive panel of hemostatic markers, including functional tests, to assess their predictive value for clinical outcomes in post-TAVR patients. The strength of this study lies in its assessment of functional hemostatic markers, allowing differentiation between platelet aggregation and fibrin coagulation. The main limitation is the small sample size, which reduces the statistical power of the findings. Blood samples were collected before discharge whenever possible, but variations in post-TAVR conditions may have affected hemostatic parameters.

## Conclusion

Selected hemostatic markers, with functional fibrinogen as a key contributor, associated with MACE within 6 months in post-TAVR patients**.** High on-treatment platelet reactivity to ADP associated with a lower risk of major or non-major clinically relevant bleeding in TAVR patients**.** Differentiating between platelet aggregation and fibrin coagulation could be a valuable focus for future trials to enhance personalized antithrombotic therapy.

## Supplementary Information

Below is the link to the electronic supplementary material.

ESM 1 (DOCX 182 KB)

## Data Availability

The datasets used and/or analyzed during the current study are available from the corresponding author upon reasonable request.
