# Supplementary material for: Fibrin clot strength is associated with increased risk of major adverse cardiac events after TAVR
Source: Clin Res Cardiol. 2025 Oct 6;114(11):1606–15. doi: 10.1007/s00392-025-02749-7 (PMC12540584; doi:10.1007/s00392-025-02749-7)
Supplement: Supplementary file 1 — (DOCX 182 KB) [file 392_2025_2749_MOESM1_ESM.docx]

**Supplemental**

**Supplemental Table S1: Inclusion and exclusion criteria**

| **Inclusion criteria** | - Informed consent - ≥18 years of age |
| --- | --- |
| **Exclusion criteria** | - GFR (MDRD) <30ml/min/173m² - Inherited coagulopathy (such as hemophilia A or B, von Willebrand disease, rare factor deficiencies) - Any other condition as determined by the PI. (e.g. incompliance, drug abuse, unwillingness to adhere to the protocol) - Valve-in-Valve transcateter aortic valve replacement |

**Table S2. Clinical Baseline Characteristics**

| Baseline characteristics | Total n=107 |
| --- | --- |
| Procedural characteristics | |
| Edwards Sapien 3  20mm  23mm  26mm  29mm | 93 (87%)  4 (4%)  32 (30%)  41 (38%)  16 (15%) |
| Medtronic Evolut R  23mm  26mm  29mm  34mm | 14 (13%)  1 (1%)  4 (4%)  8 (8%)  1 (1%) |
| Antithrombotic therapy at discharge | |
| ASA mono  DAPT  Clopidogrel mono | 22 (21%)  33 (31%)  3 (3%) |
| ASA + OAC  DAPT + OAC  P2Y12 Inhibitor + OAC  OAC mono | 29 (27%)  12 (11%)  4 (4%)  4 (4%) |
| Antithrombotic therapy at follow-up | |
| ASA mono therapy  DAPT  Clopidogrel | 31 (29%)  16 (15%)  1 (1%) |
| ASA + OAC  P2Y12 Inhibitor + OAC  OAC mono | 5 (5%)  10 (9%)  41 (38%) |
| Not Known | 3 (3%) |
| Values expressed in median (Interquartile range [IQR]) or n (%).  Abbreviations: ASA, acetylsalicylic acid; DAPT, Dual antiplatelet therapy; OAC, Oral anticoagulation | |

**Table S3. Secondary outcomes**

| *Secondary outcome* | Total n=104 |
| --- | --- |
| Hospitalized patients  Reason for rehospitalization  Procedure/valve related  Other cardiovascular  Heart failure  Non-cardiovascular  Hospitalized > 1 time | 32 (31%)  1 (1%)  13 (13%)  5 (5%)  18 (17%)  5 (5%) |
| Values expressed in n (%). | |

**Table S4. Hemostatic parameters**

| Functional hemostatic parameters | Median (IQR) |
| --- | --- |
| TEG, Global hemostasis |  |
| R-CK [min] | 7.4 (6.7-8.3) |
| R-CKH [min] | 7.2 (6.5-8.3) |
| R-HKH [min] | 5.2 (4.35-5.8) |
| alpha-CRT [degree] | 78.5 (77.1-79.85) |
| MA-CK [mm] | 65.3 (62.25-66.9) |
| MA-CKH [mm] | 65 (61.95-68.25) |
| MA-HKH [mm] | 65.8 (63.9-68.7) |
| MA-CRT [mm] | 66.6 (64.2-69.2) |
| MA-CFF [mm] | 30.9 (25.05-37.8) |
| a10-CFF [mm] | 28.8 (24.2-35) |
| LY30-CK [%] | 0.7 (0.1-1.5) |
| LY30-CRT [%] | 0.1 (0-0.4) |
| TEG, Platelet mapping |  |
| AA-MA [mm] | 35 (20.3-53.55) |
| ADP-MA [mm] | 57.7 (53-62.35) |
| ActF-MA [mm] | 19.9 (17.6-23.2) |
| LTA |  |
| MA-TRAP [%] | 80 (72-90) |
| MA-AA [%] | 20.5 (13-51.5) |
| FA-AA [%] | 17.5 (10-48.5) |
| DA-AA [%] | 0 (0-4) |
| PA-AA [%] | 20.5 (13-51.5) |
| FA-ADP [%] | 63.5 (38-75.25) |
| MA-ADP [%] | 68 (56-79) |
| DA-ADP [%] | 5 (0-16) |
| PA-ADP [%] | 68 (56-79) |
| **Laboratory parameters** |  |
| Thrombocytes [tsd/µl] | 165 (131-227.5) |
| Immature platelets [tsd/µl] | 7.35 (5.425-9.875) |
| INR | 1.05 (1.005-1.12) |
| aPTT [s] | 31 (29-35) |
| Anti Xa [ng/ml] | 108 (53.25-187.5) |
| Hämoglobin [g/dl] | 10.7 (9.5-12.5) |
| Hämatokrit [%] | 32 (28.2-37.25) |
| Vwf antigen [%] | 204 (162-257) |
| Vwf activity [%] | 195 (147-265) |
| Phospholipid IgG [U/ml] | 4 (3-6) |
| Beta2 Glykoprotein [E/ml] | 2 (1-2) |
| Phospholipid IgM [U/ml] | 2 (2-4) |
| D-Dimer [mg/l] | 1.56 (0.94-2.255) |
| Fibrinogen [mg/dl] | 461 (413.5-538) |
| Protein C [%] | 90 (80-101.5) |
| Protein S [%] | 78 (68-92.9) |
| Faktor XIII [%] | 120 (104.5-136.25) |
| Antithrombin III [%] | 92 (82-103) |
| Creatinine [mg/dl] | 0.96 (0.785-1.17) |
| GPT [U/l] | 22 (15-30.5) |
| GOT [U/l] | 30 (23-39.5) |
| AP [U/l] | 79.5 (63-97.75) |
| Leucocytes [tsd/µl] | 8.01 (6.765-9.45) |
| CRP [mg/dl] | 32.3 (16.3-53.45) |
| Parameters expressed in median (Interquartile range, IQR). |  |

**Figure S1. Clinical characteristics as predictors for MACE and bleeding**

**Figure S1,**. A: COX regression with clinical characteristics as predictors for the occurrence of MACE within 6 months B: COX regression with clinical characteristics as predictors for the occurrence of bleeding within 6 months. MACE: Major adverse cardiac event, HR: Hazard ratio, BMI: Body mass index, TTE: Transthoracic echocardiography. T0: measurement during hospitalization

**Figure S2. Functional hemostatic markers as predictors for Bleeding. Subgroup analysis of patients with ADP-receptor antagonists**

**Figure S2**. Cox regression of functional hemostatic markers as predictors for the occurrence of bleeding within 6 months. Subgroup analysis of patients with ADP-receptor antagonists (n=34). Bleeding: Major and nonmajor clinical relevant bleeding, TEG: Thrombelastography, PM: platelet mapping, LTA: Light transmission aggregometry MA: Maximum aggregation, ADP: Adenosin diphosphate, , FA: final aggregation, DA: Disaggregation, PA: Primary aggregation.

**Figure S3** **Kaplan Meier for non-procedure related Bleeding**

| **Patients at Risk** | 107 | 106 | 101 | 99 | 98 | 96 | 96 |
| --- | --- | --- | --- | --- | --- | --- | --- |
